# Supplementary material for: Pulse potential mediated selectivity for the electrocatalytic oxidation of glycerol to glyceric acid
Source: Nat Commun. 2024 Mar 18;15:2420. doi: 10.1038/s41467-024-46752-4 (PMC10948758; doi:10.1038/s41467-024-46752-4)
Supplement: Supplementary file 1 — Supplementary Information [file 41467_2024_46752_MOESM1_ESM.pdf]

Supporting Information

Pulse potential mediated selectivity for the electrocatalytic oxidation of glycerol to glyceric acid

Wei Chen[1,2,†], Liang Zhang[1,3, †], Leitao Xu[1,†], Yuanqing He[1,2], Huan Pang[2 ✉], Shuangyin Wang[1] & Yuqin Zou[1✉]

[1] State Key Laboratory of Chem/Bio-Sensing and Chemometrics, College of Chemistry and Chemical Engineering, Hunan University, Changsha, 410000, P. R. China.
[2] School of Chemistry and Chemical Engineering, Yangzhou University, Yangzhou, 225009, P. R. China.
[3] Key Laboratory of Leather of Zhejiang Province, Institute of New Materials and Industrial Technologies, Wenzhou University, Wenzhou, Zhejiang 325035, P. R. China
[†] These authors contributed equally: Wei Chen, Liang Zhang, Leitao Xu.

✉email: panghuan@yzu.edu.cn; yuqin_zou@hnu.edu.cn

Table of contents

I. Supplementary Figures 3

Supplementary Fig. 1 3

Supplementary Fig. 2 4

Supplementary Fig. 3 5

Supplementary Fig. 4 6

Supplementary Fig. 5 7

Supplementary Fig. 6 8

Supplementary Fig. 7 9

Supplementary Fig. 8 10

Supplementary Fig. 9 11

Supplementary Fig. 10 12

Supplementary Fig. 11 13

Supplementary Fig. 12 14

Supplementary Fig. 13 15

Supplementary Fig. 14 16

Supplementary Fig. 15 17

Supplementary Fig. 16 18

Supplementary Fig. 17 19

Supplementary Fig. 18 20

Supplementary Fig. 19 21

Supplementary Fig. 20 22

Supplementary Fig. 21 23

Supplementary Fig. 22 24

Supplementary Fig. 23 25

II. Supplementary Tables 26

Supplementary Table 1 26

Supplementary Table 2 27

Supplementary Table 3 28

Supplementary Table 4 29

Supplementary Table 5 30

III. Supplementary References 31

I. Supplementary Figures

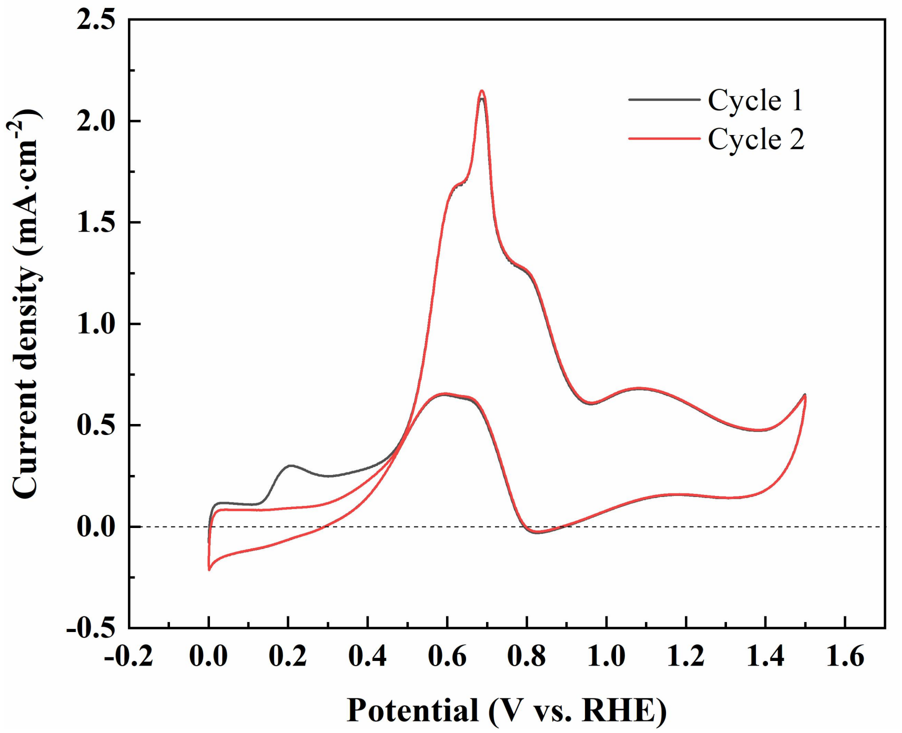

Supplementary Fig. 1 The two cycles of CV curves of Pt@G with 20 mM glycerol.

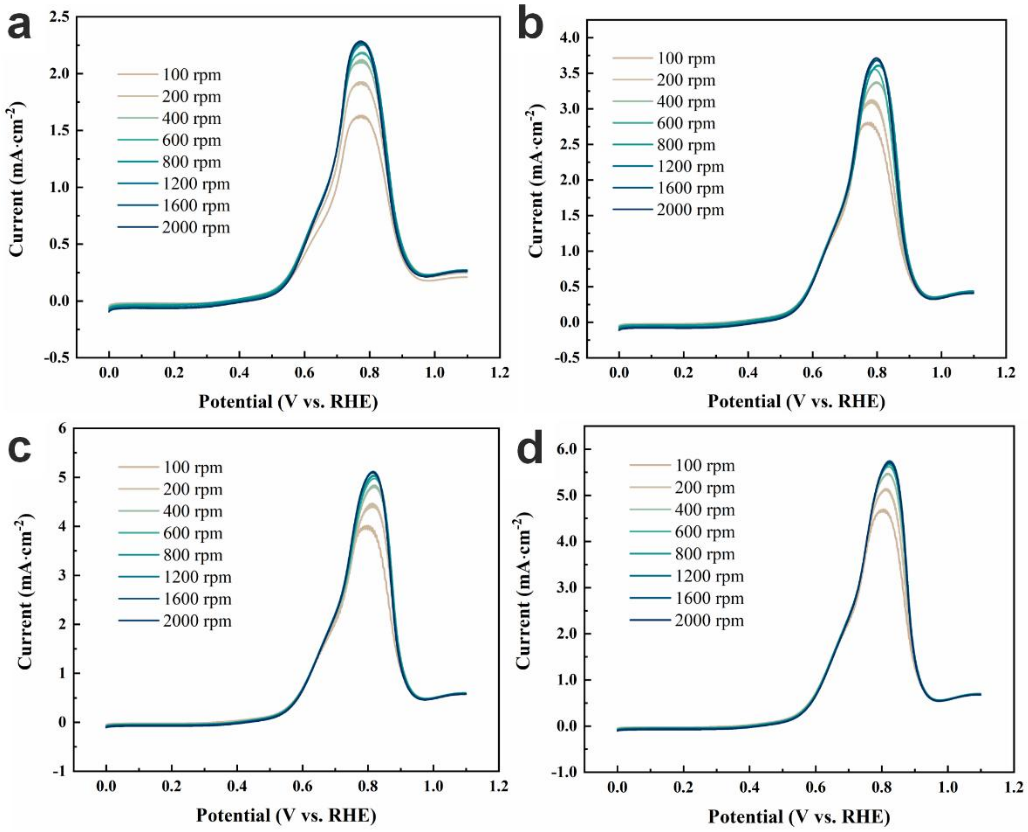

Supplementary Fig. 2 The LSV curves of Pt@G using rotating disc electrodes at various rotation speeds with different glycerol concentrations. (a) 20 mM; (b) 40 mM; (c) 60 mM; (d) 80 mM.

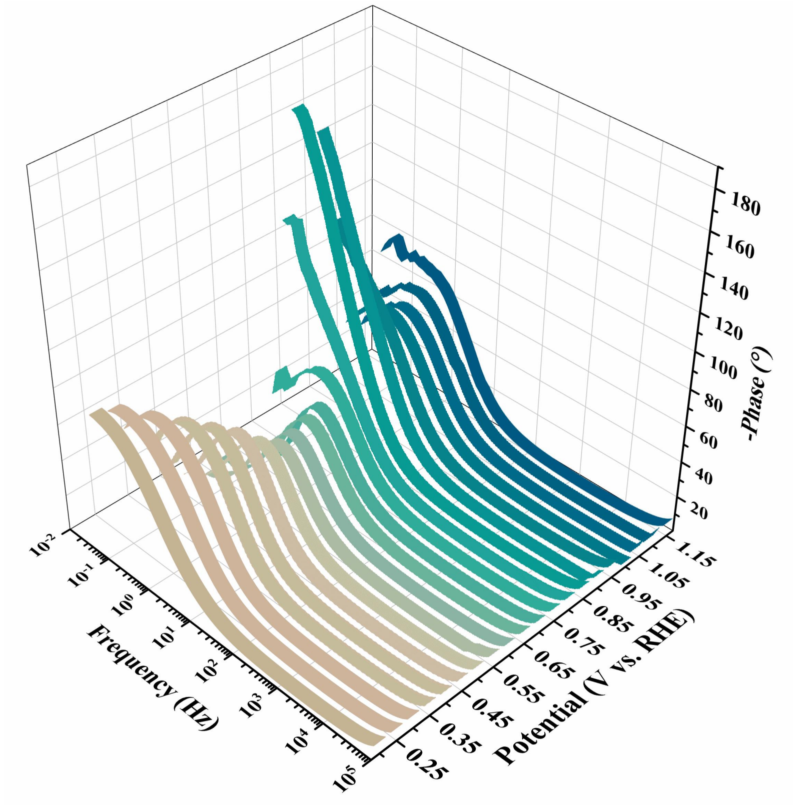

Supplementary Fig. 3 Bode plots of Pt@G with 50 mM glycerol at various potentials.

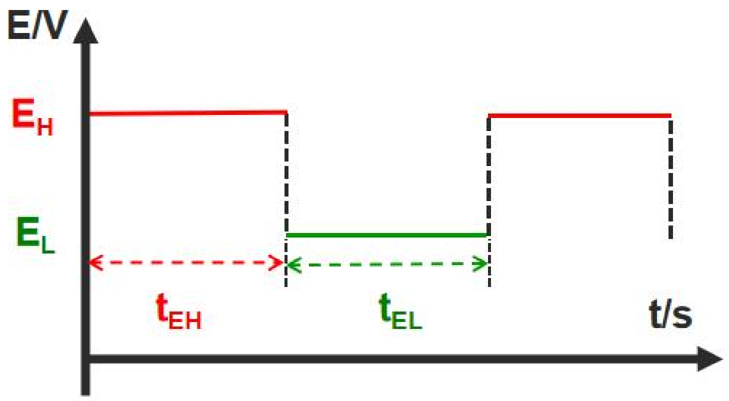

Supplementary Fig. 4 The schematic of pulsed potential electrocatalyst protocol.

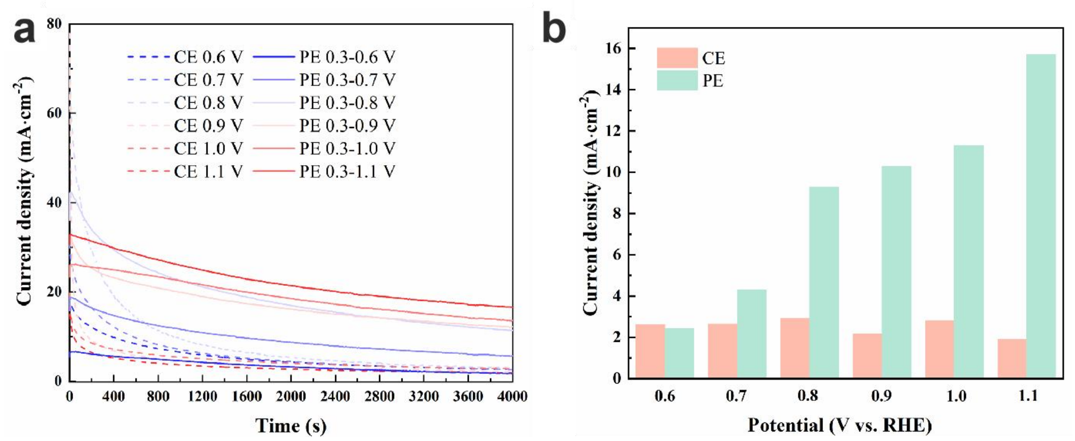

Supplementary Fig. 5 (a) Current density comparison of CE and PE with 50 mM glycerol in 1 M KOH for 4000 s. (b) The current density of CE and PE protocol with different potentials after 4000 s electrolysis.

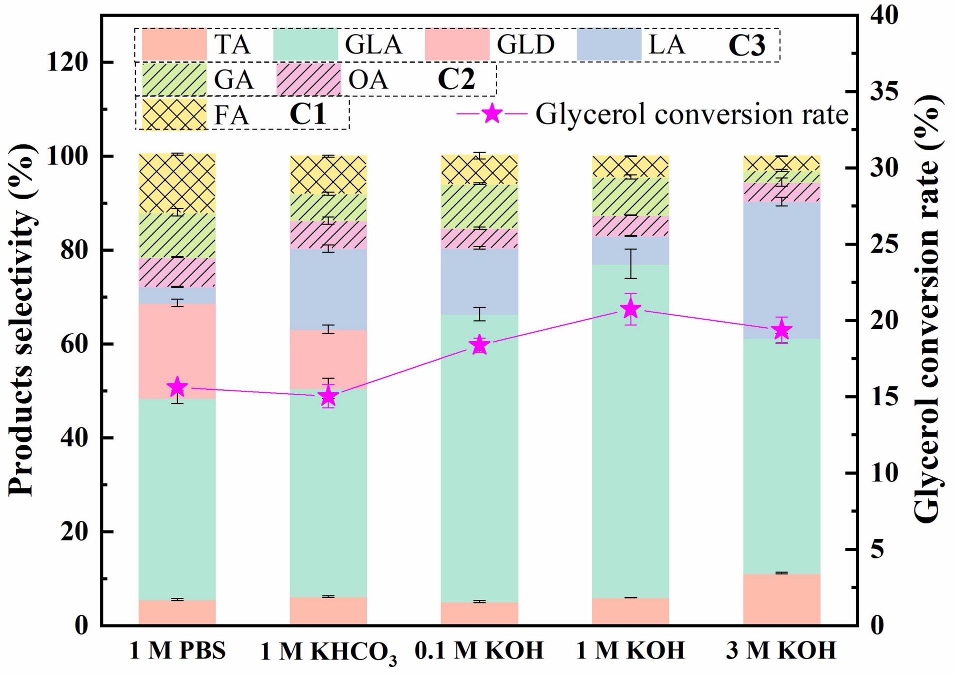

Supplementary Fig. 6 The selectivity and glycerol conversion rate with 50 mM glycerol of PE (E_L = 0.3 V_{RHE}, E_H =0.7 V_{RHE}, t_{EL}=0.5 s, t_{EH}=0.05 s) in different electrolytes with a same charge of 20 C. TA = tartronic acid, GLA = glyceric acid, GLD = glyceraldehyde, LA = lactic acid, OA = oxalic acid, GA = glycolic acid, FA=formic acid.

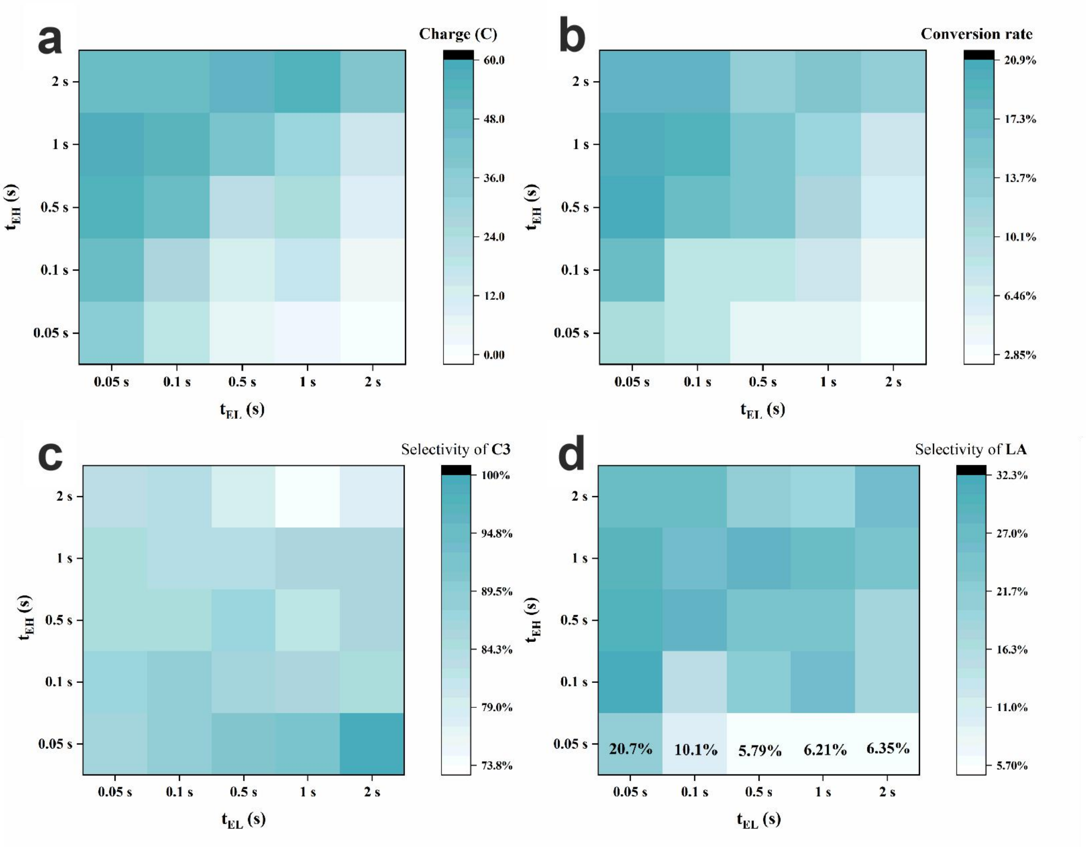

Supplementary Fig. 7 The conditions optimization of t_{EL} and t_{HE} (E_L = 0.3 V_{RHE}, E_H =0.7 V_{RHE}, 50 mM GLY, electrolysis time is 1 hour). (a) Coulomb volume; (b) conversion rate of glycerol; (c) selectivity of C3; (d) selectivity of lactic acid (LA).

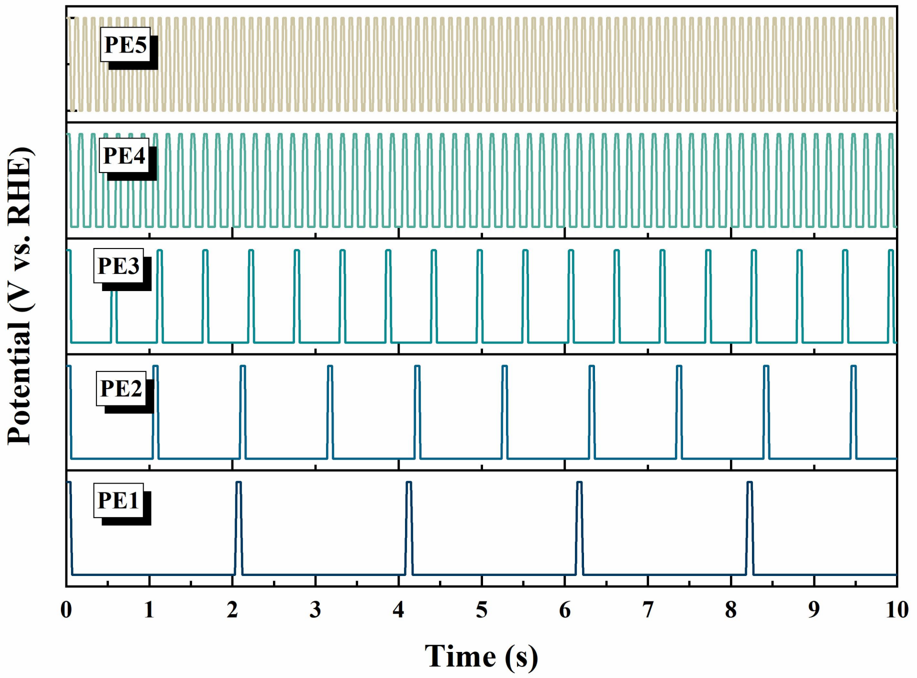

Supplementary Fig. 8 The schematic of different PE protocols. E_H =0.7 V_{RHE} for 0.05 s, E_L = 0.3 V_{RHE} for 2.0 s (PE1), 1.0 s (PE2), 0.5 s (PE3), 0.1 s (PE4) and 0.05 s (PE5).

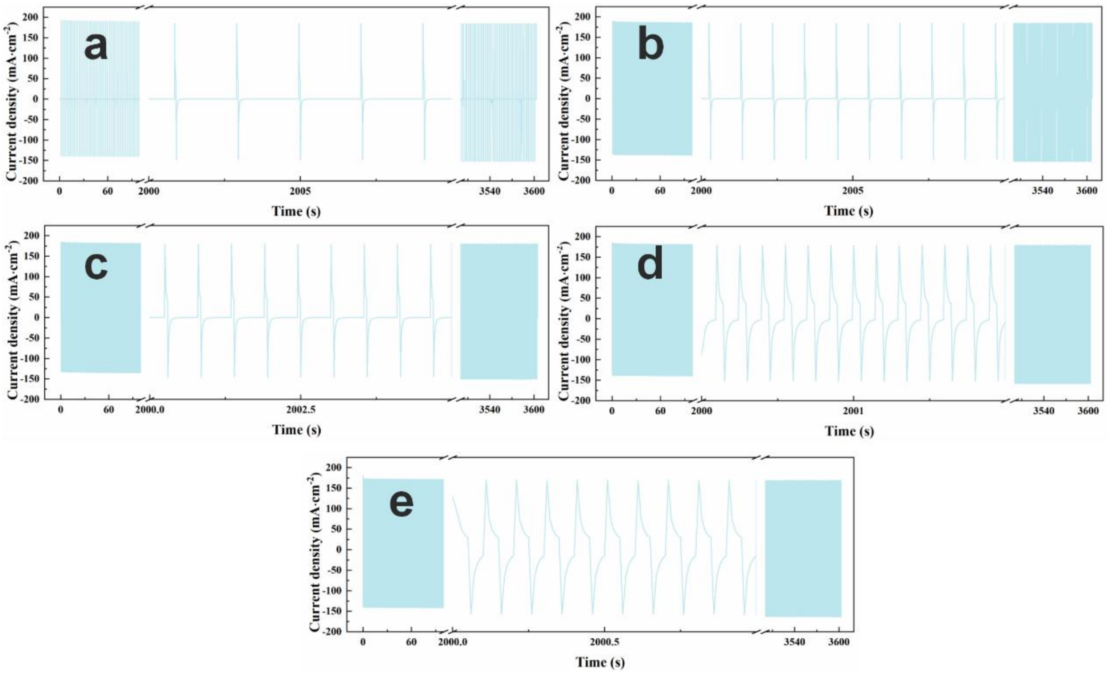

Supplementary Fig. 9 The I-t curves of Pt@G using the pulsed potential electrolysis with 50 mM glycerol. (a) PE1; (b) PE2; (c) PE3; (d) PE4; (e) PE5.

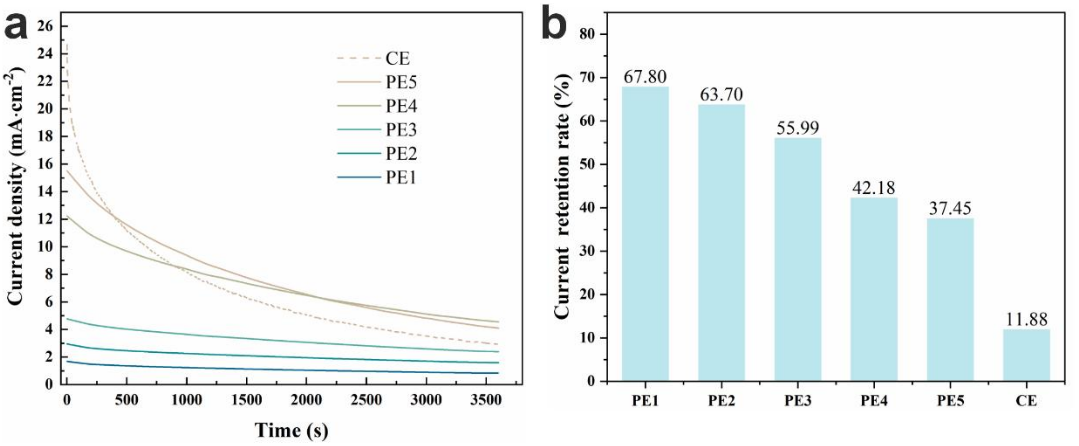

Supplementary Fig. 10 The current density (a) and its retention rate (b) of CE (0.7 V_{RHE}) and different PE protocols (PE1-PE5).

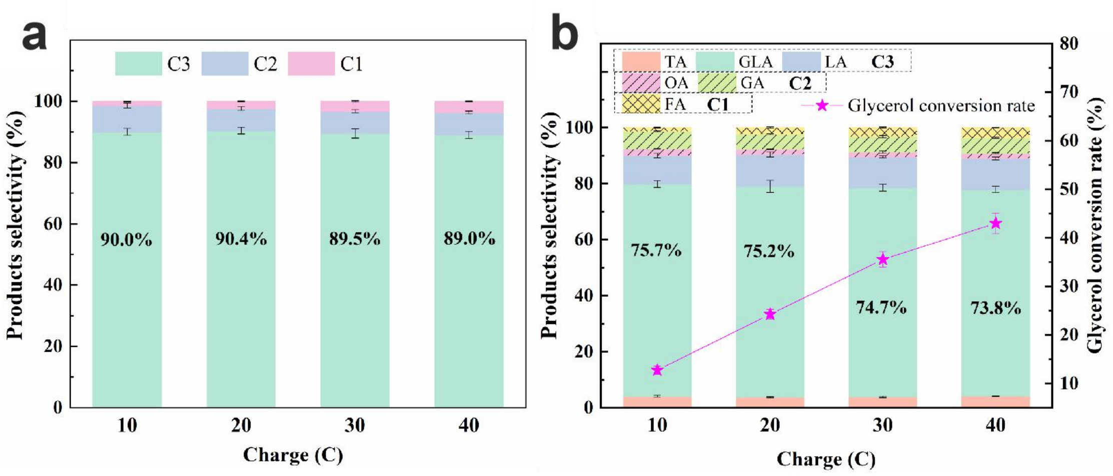

Supplementary Fig. 11 The selectivity and glycerol conversion rate with 50 mM glycerol of PE3. The percentages in black indicate the selectivity of **a** C3 and **b** GLA. TA = tartronic acid, GLA = glyceric acid, LA = lactic acid, OA = oxalic acid, GA = glycolic acid, FA=formic acid.

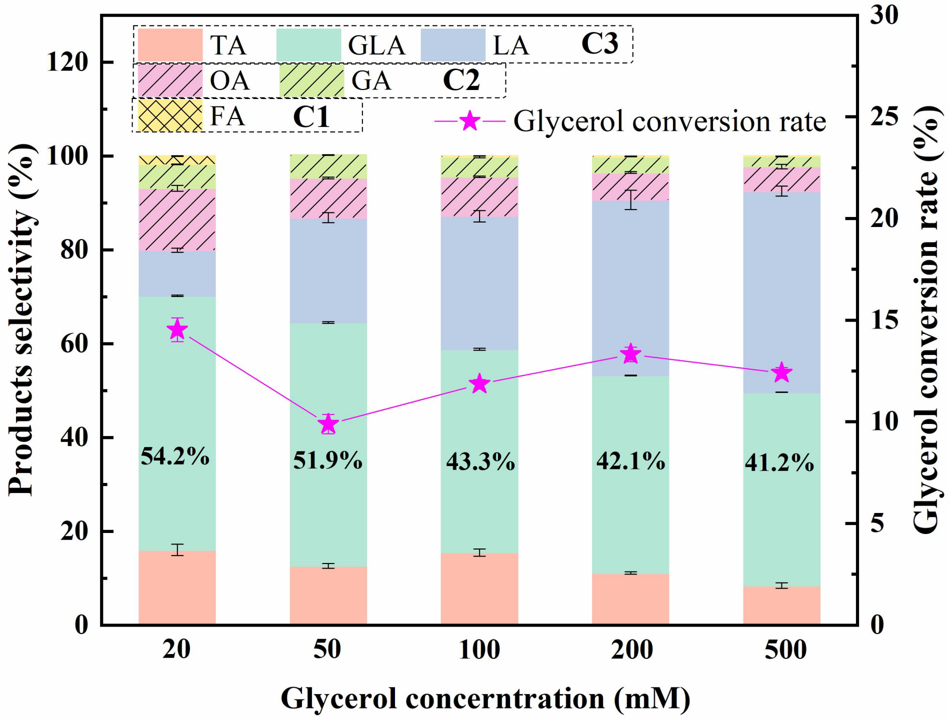

Supplementary Fig. 12 The selectivity and glycerol conversion rate of PE5 with different glycerol concentrations. The percentages in black indicate the selectivity of GLA. TA = tartronic acid, GLA = glyceric acid, LA = lactic acid, OA = oxalic acid, GA = glycolic acid, FA=formic acid.

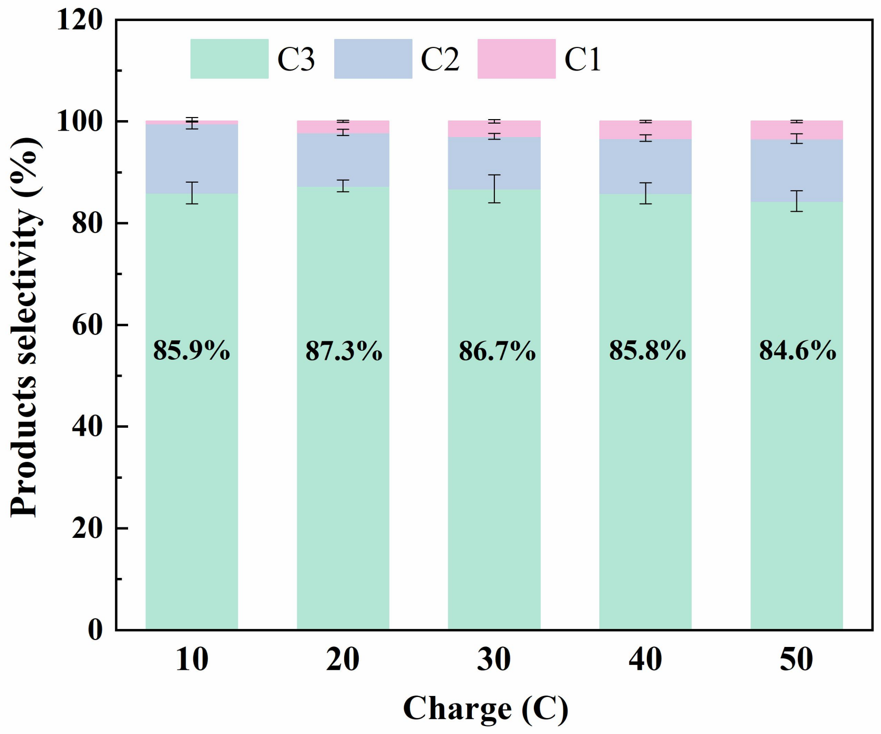

Supplementary Fig. 13 The selectivity of C1, C2 and C3 of PE3 with 20 mM of glycerol. The percentages in black indicate the selectivity of C3.

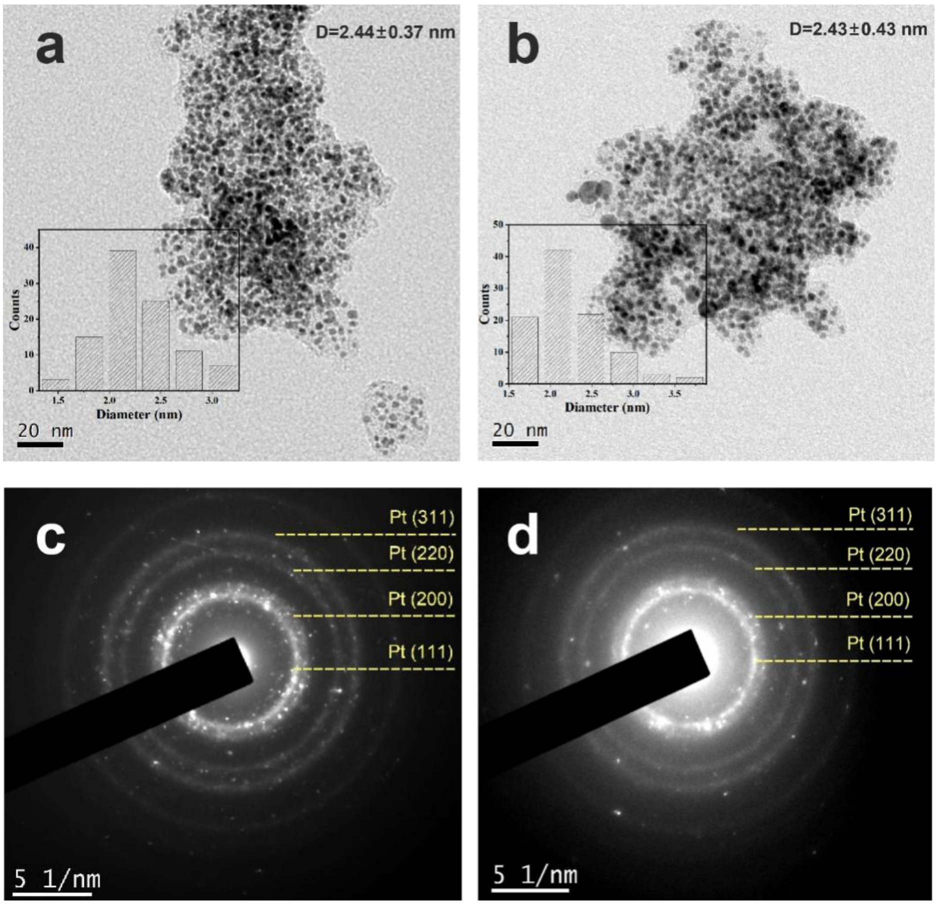

Supplementary Fig. 14 TEM images of (a) Pt@G after PE, and (b) Pt@G after CE and SAED patterns of (c) Pt@G after PE, and (d) Pt@G after CE. **a**, **b** Inset shows the particle size distribution and its average value of Pt@G nanoparticles. **c, d** Interpolated text marks the different crystal planes of Pt.

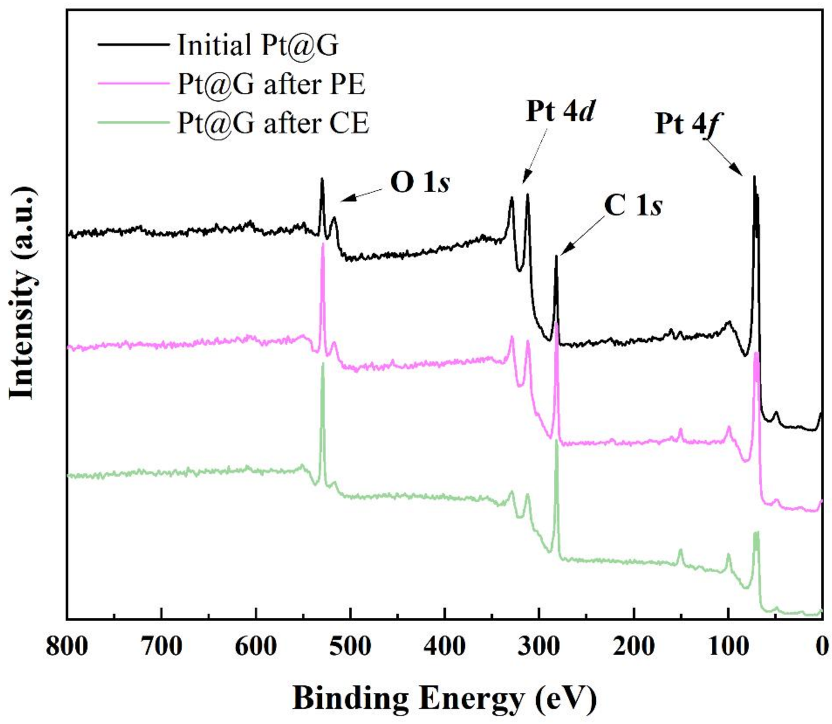

Supplementary Fig. 15 XPS analysis of different Pt@G catalysts.

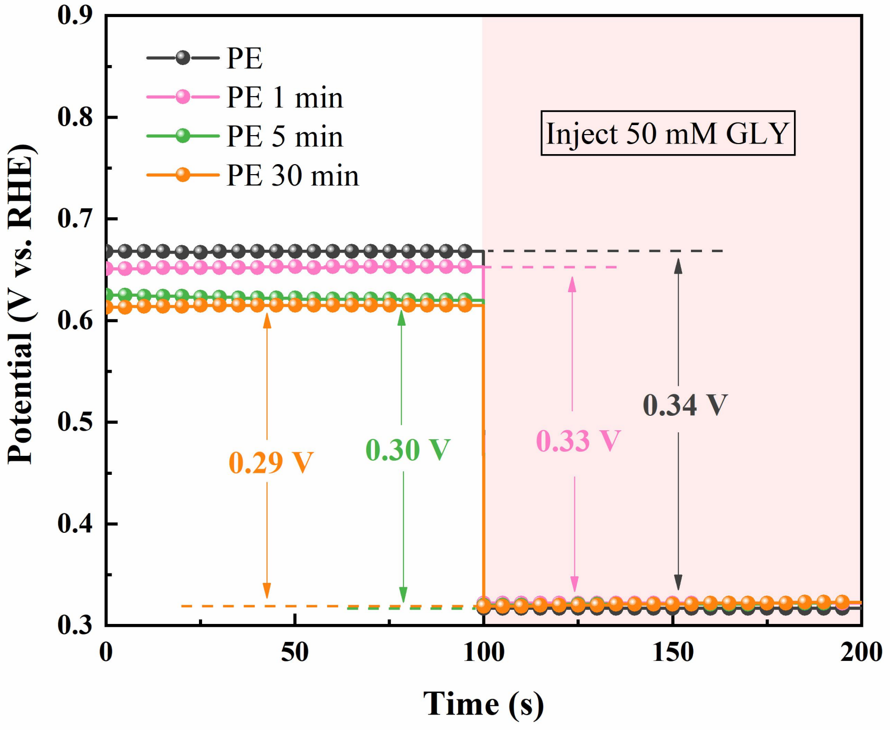

Supplementary Fig. 16 OCP curves of Pt@G after PE (E_L = 0.3 V_{RHE}, E_H =0.7 V_{RHE}, t_{EL}=t_{EH}=0.5 s) with different times with 50 mM glycerol being injected subsequently. The numbers in the figure indicate the value of the potential (vs. RHE) difference of the OCP.

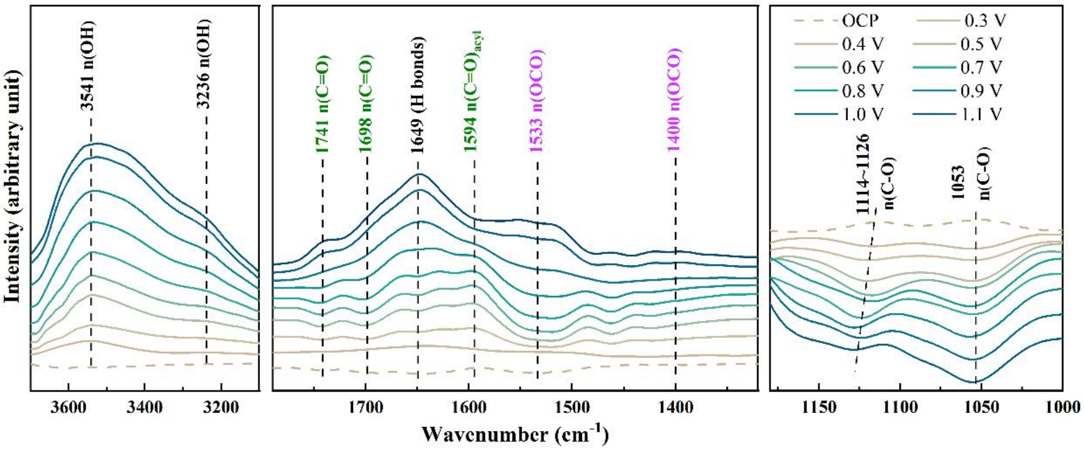

Supplementary Fig. 17 *In situ* FTIR over Pt@G catalysts with 50 mM glycerol at different potentials.

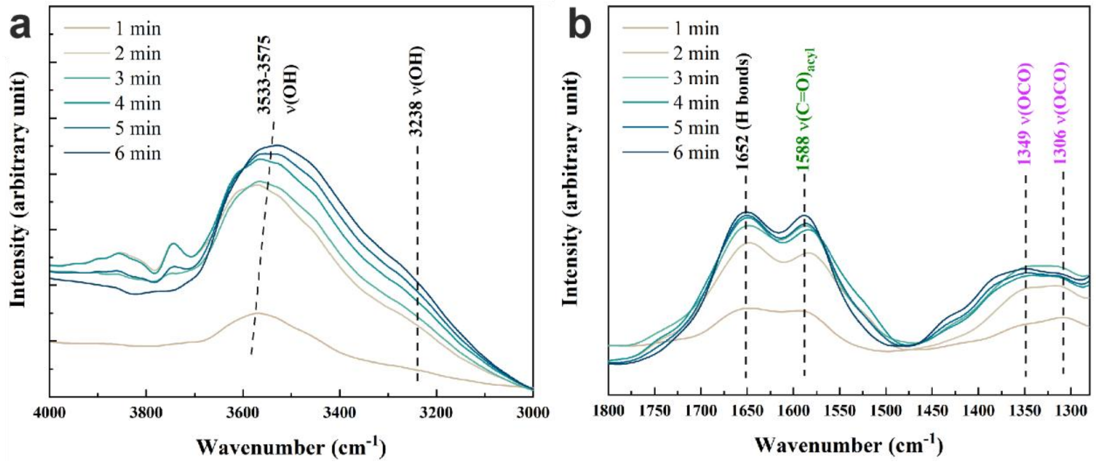

Supplementary Fig. 18 *In situ* FTIR over Pt@G catalysts with 50 mM glycerol at 1.1 V_{RHE} for different times.

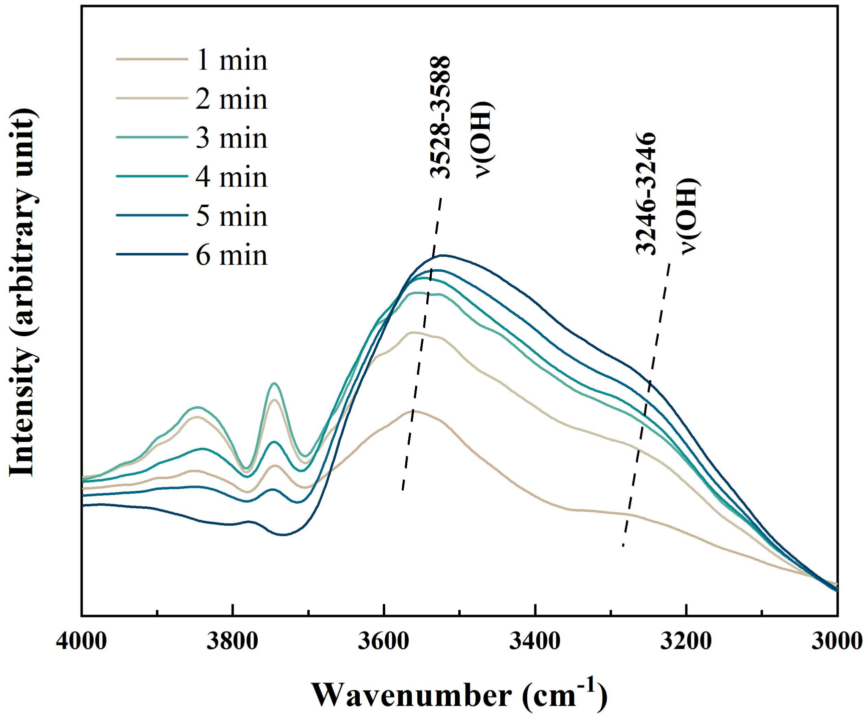

Supplementary Fig. 19 *In situ* FTIR over Pt@G catalysts with 50 mM glycerol at 0.7 V_{RHE} for different times.

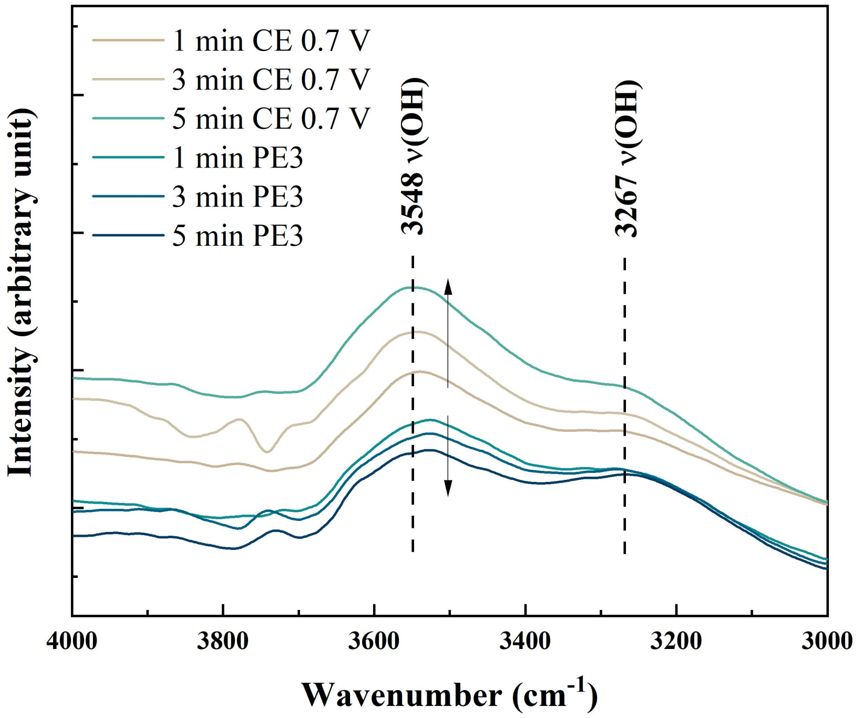

Supplementary Fig. 20 *In situ* FTIR over Pt@G catalysts with 50 mM glycerol at 0.7 V_{RHE} and PE3 for different times.

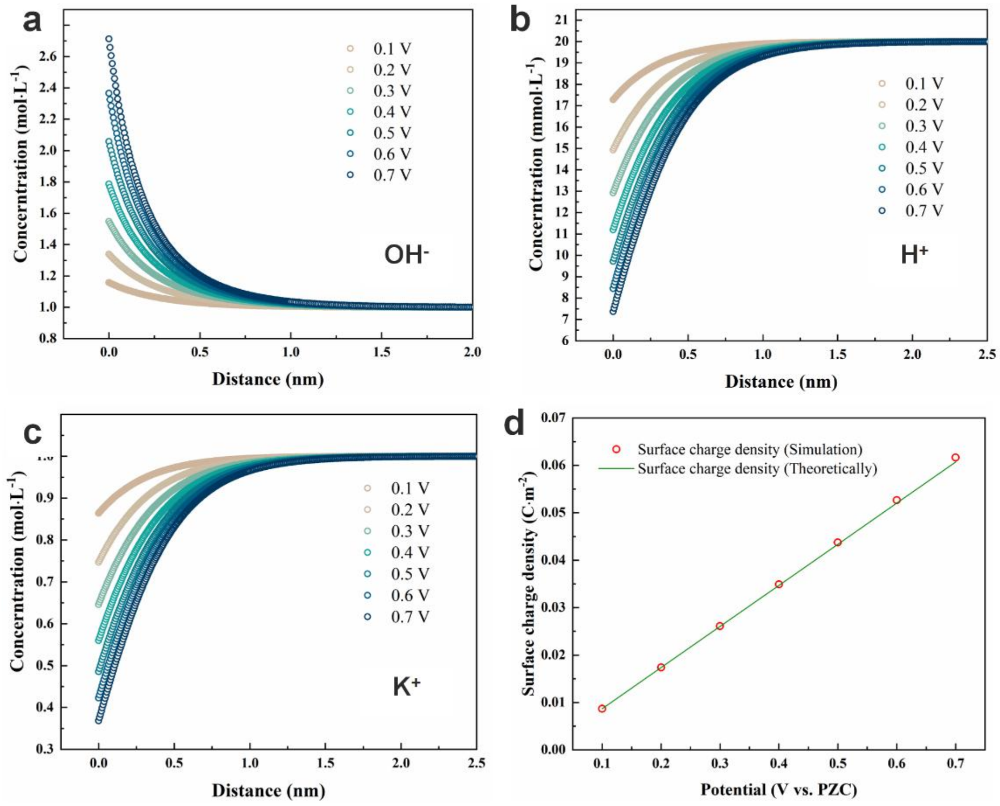

Supplementary Fig. 21 Effect of applied potential on the diffusion of (a) OH^-, (b) H^+ and (c) K^+ on the electrode surface. (d) The correlation of simulation and theoretical surface charge.

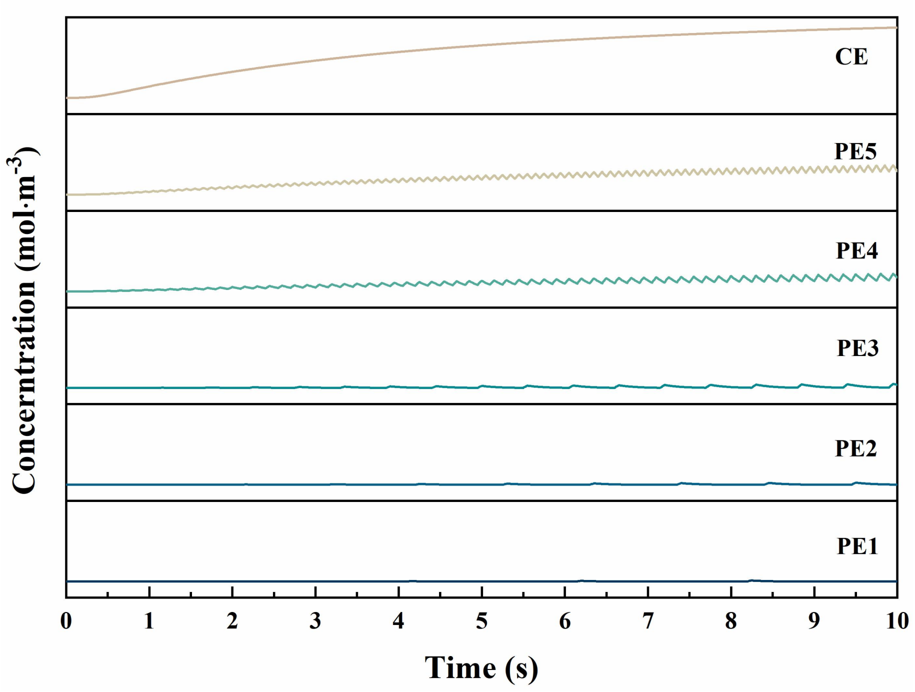

Supplementary Fig. 22 The surface concentration of glyceric acid at different electrolysis times for CE and PE1-PE5.

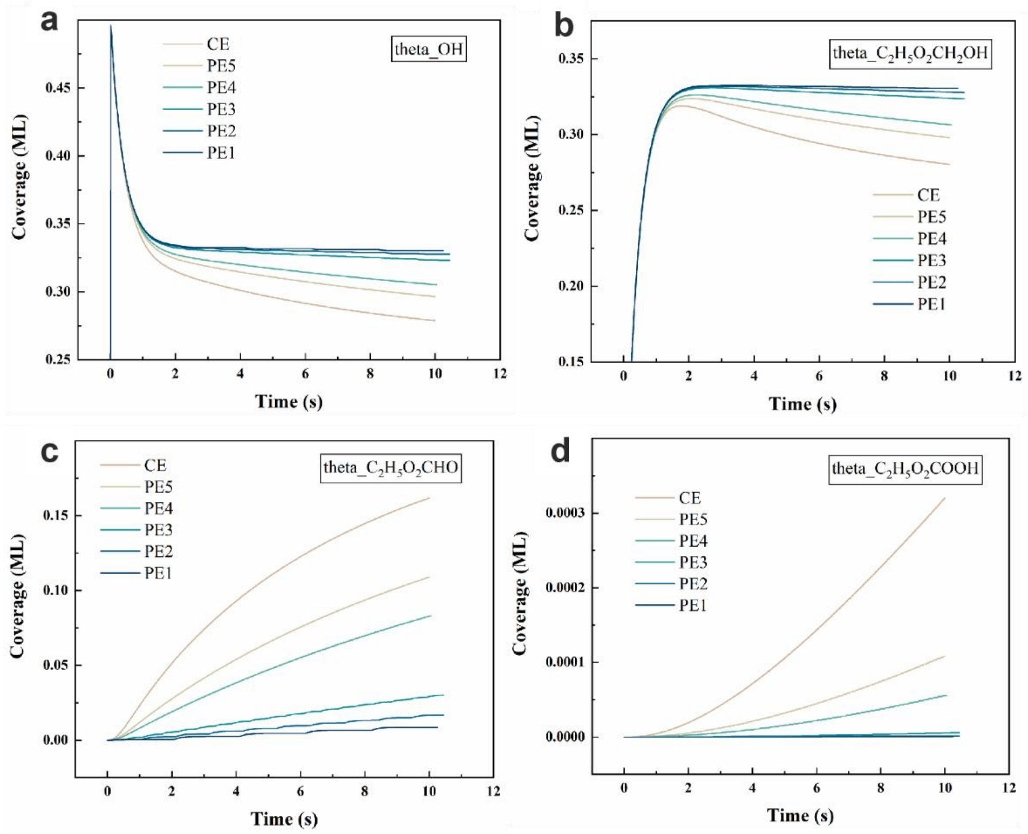

Supplementary Fig. 23 The time-depends surface coverage of different spices. (a) OH^-, (b) $C_2H_5O_2CH_2OH$, (c) $C_2H_5O_2CHO$ and (d) $C_2H_5O_2COOH$

II. Supplementary Tables

Supplementary Table 1. Main parameters for finite element simulations of the diffuse double layer.

Parameters	Value	Description
T0	298.15 K	Temperature
eps_H2O	78.5	The relative permittivity of water
xD	3.0121E-10 m	Debye length
c_OH	1000 mol/m³	Bulk anion concentration of OH^-
c_K	1000 mol/m³	Bulk anion concentration of K^+
c_C2H5O2COO	20 mol/m³	Bulk anion concentration of $C_2H_5O_2COO^-$
c_H	20 mol/m³	Bulk anion concentration of H^+
D_OH	5.2E-9 m²/s	Diffusion coefficient
D_K	1.957E-9 m²/s	Diffusion coefficient
D_C2H5O2COO	8.25E-10 m²/s	Diffusion coefficient
D_H	9.31E-9 m²/s	Diffusion coefficient

Supplementary Table 2. Main parameters for Finite element simulations of the GEOR.

**Parameters**	**Value**	**Description**
T	298.15 K	Temperature
F	96485 C/mol	Faraday constant
R	8.314 J/(mol K)	Ideal gas constant
α_a	0.5	Electron transport coefficient
α_c	0.5	Electron transport coefficient
γ	0.1 mol/m^2	Absorption spices density
c_C2H5O2CH2OHb	1000 mol/m^3	The bulk concentration of GLY
c_OH	1000 mol/m^3	Bulk anion concentration of OH^-
c_K	1000 mol/m^3	Bulk anion concentration of K^+
D_C2H5O2CH2OH	8.25E-10 m^2/s	Diffusion coefficient
D_C2H5O2CHO	8.25E-10 m^2/s	Diffusion coefficient
D_C2H5O2COOH	8.25E-10 m^2/s	Diffusion coefficient
D_OH	5.2E-9 m^2/s	Diffusion coefficient
D_K	1.957E-9 m^2/s	Diffusion coefficient
D_C2H5O2COO	8.25E-10 m^2/s	Diffusion coefficient
D_H	9.31E-9 m^2/s	Diffusion coefficient
k4_0	3e-2 m/s	Reaction rate constant
k5_0	3e-2 m/s	Reaction rate constant
k6_0	3e-2 m/s	Reaction rate constant
k7_0	3e-2 m/s	Reaction rate constant
k8_0	1.39E-06 m/s	Reaction rate constant
k9_0	1.39E-06 m/s	Reaction rate constant
K_C2H5O2COOH	2.29E-4	Ionization equilibrium constant
K_H2O	1E-14	Ionization equilibrium constant
E_{eq}	0.3 V	Equilibrium potential
C_{dl}	0.01 F/m^2	Double layer capacitor

Supplementary Table 3. Comparison of selectivity of C3 and GLY conversion rate with other reports.

Catalyst	Reaction conditions	Conversion	Selectivity& yield	Reference
Pt@G	0.05 M glycerol, 1.0 M KOH, PE 0.3-0.7 V vs. RHE	14.7~59.4%	81.8~74.4% (GLA)	This work
AuPt	0.5 M glycerol, 1.0 M KOH, 0.45 V vs. Hg/HgO	10.7%	50% (LA)	[1]
PtAg skeleton	0.5 M glycerol, 0.5 M KOH, 0.9 V vs. RHE	<5%	62% (DHA)	[2]
Pt_5Ru_5/C	0.1 M glycerol, 0.5 M H_2SO_4, 1.1 V vs. SHE	20.3%	58% (GLA)	[3]
PtRhNi/GNS	0.5 M glycerol, 1.0 M KOH, 0.4 V vs. SCE	<5%	55% (GLA)	[4]
Pd	0.1 M glycerol, 1.0 M KOH, -0.1 V vs. Hg/HgO	<1%	56% (GLA)	[5]
Pt-CeO_2/CNT	0.1 M glycerol, 1.0 M KOH, 0.9 V vs. RHE	86%	59% (GLA)	[6]
Pt/CNTs-CeO_2	0.1 M glycerol, 1.0 M KOH, 0.8 V vs. RHE	55.3%	58% (GLA)	[7]

Supplementary Table 4. The different elemental content derived from the XPS spectrum.

Catalysts	C (%)	O (%)	Pt (%)	Sum (%)
Initial Pt@G	75.42	19.56	5.01	99.99
Pt@G after PE	75.79	20.97	3.23	99.99
Pt@G after CE	73.58	24.7	1.71	99.99

Supplementary Table 5. The different Pt peak areas derived from high-resolution XPS refined spectrum.

Catalysts	Pt peak1	Pt peak2	Pt(II) peak1	Pt(II) peak2	Proportions of Pt(II)
Initial Pt@G	13670.6	14267.65	1994.259	3424.605	16.2%
Pt@G after PE	29671	33340.36	5454.317	7741.874	17.3%
Pt@G after CE	32649.31	39767.99	20097.63	8973.615	28.6%

II. Supplementary References

Reference

1. Dai C*, et al.* Electrochemical production of lactic acid from glycerol oxidation catalyzed by AuPt nanoparticles. *J Catal* **356**, 14-21 (2017).
2. Zhou Y, Shen Y, Xi J, Luo X. Selective Electro-Oxidation of Glycerol to Dihydroxyacetone by PtAg Skeletons. *ACS Appl Mater Interfaces* **11**, 28953-28959 (2019).
3. Kim Y*, et al.* The Role of Ruthenium on Carbon-Supported PtRu Catalysts for Electrocatalytic Glycerol Oxidation under Acidic Conditions. *ChemCatChem* **9**, 1683-1690 (2017).
4. Zhou Y, Shen Y, Piao J. Sustainable Conversion of Glycerol into Value-Added Chemicals by Selective Electro-Oxidation on Pt-Based Catalysts. *ChemElectroChem* **5**, 1636-1643 (2018).
5. Inoue H, Kimura S, Teraoka Y, Chiku M, Higuchi E, Lam BTX. Mechanism of glycerol oxidation reaction on silver-modified palladium electrode in alkaline medium. *Int J Hydrogen Energy* **43**, 18664-18671 (2018).
6. Li J*, et al.* Tuning the Product Selectivity toward the High Yield of Glyceric Acid in Pt−CeO2/CNT Electrocatalyzed Oxidation of Glycerol. *ChemCatChem* **14**, e202200509 (2022).
7. Liu X, Yang C. Electrocatalytic selective oxidation of glycerol to glyceric acid over efficient Pt/CNTs-CeO2 catalysts. *Mater Lett* **324**, 132658 (2022).
